# Uptake, Outcomes, and Costs of Antenatal, Well-Baby, and Prevention of Mother-to-Child Transmission of HIV Services under Routine Care Conditions in Zambia

**DOI:** 10.1371/journal.pone.0072444

**Published:** 2013-08-28

**Authors:** Callie A. Scott, Hari S. Iyer, Deophine Lembela Bwalya, Maximillian Bweupe, Sydney B. Rosen, Nancy Scott, Bruce A. Larson

**Affiliations:** 1 Center for Global Health and Development, Boston University, Boston, Massachusetts, United States of America; 2 Zambia Center for Applied Health Research and Development, Lusaka, Zambia; 3 Zambian Ministry of Health, Lusaka, Zambia; 4 Health Economics and Epidemiology Research Office, Wits Health Consortium, Faculty of Health Sciences, University of the Witwatersrand, Johannesburg, South Africa; 5 Department of International Health, School of Public Health, Boston University, Boston, Massachusetts, United States of America; UCL Institute of Child Health, University College London, United Kingdom

## Abstract

**Background:**

Zambia adopted Option A for prevention of mother-to-child transmission of HIV (PMTCT) in 2010 and announced a move to Option B+ in 2013. We evaluated the uptake, outcomes, and costs of antenatal, well-baby, and PMTCT services under routine care conditions in Zambia after the adoption of Option A.

**Methods:**

We enrolled 99 HIV-infected/HIV-exposed (index) mother/baby pairs with a first antenatal visit in April-September 2011 at four study sites and 99 HIV-uninfected/HIV-unexposed (comparison) mother/baby pairs matched on site, gestational age, and calendar month at first visit. Data on patient outcomes and resources utilized from the first antenatal visit through six months postpartum were extracted from site registers. Costs in 2011 USD were estimated from the provider’s perspective.

**Results:**

Index mothers presented for antenatal care at a mean 23.6 weeks gestation; 55% were considered to have initiated triple-drug antiretroviral therapy (ART) based on information recorded in site registers. Six months postpartum, 62% of index and 30% of comparison mother/baby pairs were retained in care; 67% of index babies retained had an unknown HIV status. Comparison and index mother/baby pairs utilized fewer resources than under fully guideline-concordant care; index babies utilized more well-baby resources than comparison babies. The average cost per comparison pair retained in care six months postpartum was $52 for antenatal and well-baby services. The average cost per index pair retained was $88 for antenatal, well-baby, and PMTCT services and increased to $185 when costs of triple-drug ART services were included.

**Conclusions:**

HIV-infected mothers present to care late in pregnancy and many are lost to follow up by six months postpartum. HIV-exposed babies are more likely to remain in care and receive non-HIV, well-baby care than HIV-unexposed babies. Improving retention in care, guideline concordance, and moving to Option B+ will result in increased service delivery costs in the short term.

## Introduction

Despite current efforts to prevent mother-to-child transmission of HIV in Zambia, vertical transmission has recently been estimated at 20%, with 16,000 children newly infected with HIV in 2010 [1]. Zambia is one of 22 focus countries in the UNAIDS global plan towards the elimination of new HIV infections among children, with the goal of reducing mother-to-child transmission to less than 5% by 2015 [1], [2].

To achieve this goal, the World Health Organization (WHO) in 2010 revised its guidelines for the prevention of mother-to-child transmission of HIV (PMTCT) [3]. The WHO guidelines recommend immediate lifelong, triple-drug antiretroviral therapy (ART) for pregnant women with a CD4≤350 cells/µL or WHO clinical stage 3 or 4 disease. For pregnant women not meeting these criteria, the WHO guidelines offer two options: Option A and Option B. Under Option A, women receive ARV prophylaxis including daily zidovudine from 14 weeks gestation through one week postpartum, single-dose nevirapine at delivery, and daily lamivudine from delivery through one week postpartum. Their babies receive daily nevirapine from birth through one week after the cessation of breastfeeding. Under Option B, all women receive triple-drug ART from 14 weeks gestation through the cessation of breastfeeding. Their babies receive daily nevirapine from birth through six weeks of age. A third option, Option B+, was added to the WHO guidelines in 2012 [4]. Under Option B+, all pregnant women are eligible to initiate lifelong, triple-drug ART at their first antenatal visit, regardless of CD4, WHO clinical stage, or gestational age and their babies receive daily nevirapine as under Option B.

Zambia revised its national PMTCT guidelines in 2010 in accordance with the new WHO guidelines and, along with many other developing countries, adopted Option A [5]. In 2013, after more than two years implementing Option A, Zambia announced that it would revise its PMTCT guidelines again and adopt Option B+ [6].

Most published evidence on the implementation of PMTCT in Zambia and other countries in sub-Saharan Africa pertains to previous, less effective regimens [7], [8], [9], [10], [11], [12], [13]. Recently published papers on the cost-effectiveness of PMTCT in Nigeria, Malawi, Zimbabwe, and Rwanda draw data from multiple diverse sources and use models to estimate costs [14], [15], [16], [17]. No published papers describe antenatal, well-baby, and PMTCT service delivery and associated costs under routine care conditions, with primary patient-level data, after the adoption of either Option A or Option B+. To provide information needed to assess the implementation of Option A, and to prepare for the implementation of Option B+, we evaluated the uptake, outcomes, and costs of antenatal, well-baby, and PMTCT service delivery under routine care conditions in Zambia using primary patient-level data.

## Methods

### Ethics Statement

The Boston University Medical Center Institutional Review Board and the University of Zambia Biomedical Research Ethics Committee provided ethical approval of the study (protocol numbers H-31608 and 005-05-12). A waiver of informed consent was granted by both committees because the study was a retrospective review of routinely collected information from patient registers.

### Analytic Overview

We evaluated the uptake, outcomes, and costs of antenatal, well-baby, and PMTCT-related care for a cohort of HIV-infected mothers and their HIV-exposed babies (the index group) and a matched cohort of HIV-uninfected mothers and their HIV-unexposed babies (the comparison group) who sought care after the adoption of Option A for PMTCT in Zambia. Using retrospective patient-level data on outcomes and resource utilization and site-level data on unit costs, we estimated: (1) the proportion of index and comparison mother/baby pairs retained in care through delivery and six months after delivery; (2) the average quantity of resources utilized for antenatal, well-baby, and PMTCT-related care; and (3) the average cost of providing antenatal, well-baby, and PMTCT-related care from the first antenatal visit through six months after delivery under routine care conditions. Antenatal and well-baby care included services provided to both index and comparison mother/baby pairs before and after delivery. PMTCT-related care included services provided to index mother/baby pairs only, including ARV prophylaxis for mothers not yet on triple-drug ART, ARV prophylaxis for babies, co-trimoxazole prophylaxis for mothers and babies, and HIV DNA PCR tests for babies. PMTCT-related care did not include triple-drug ART services. In secondary analyses, we estimated the average cost of triple-drug ART services, in addition to the cost of antenatal, well-baby, and PMTCT services, the average cost of switching from Option A to Option B+, and the cost of guideline-concordant care. Costs were calculated from the provider’s perspective and are reported in 2011 US dollars.

### Study Sites

We purposively selected four public sector clinics in Southern Province in Zambia after conducting an assessment at each site to confirm that patients could be traced longitudinally through the site registers. All four study sites are run by the Government of Zambia and receive technical support for the provision of PMTCT-related care from the Boston University PMTCT Integration Project (through the President’s Emergency Plan for AIDS Relief) and other partners. Sites included an urban district hospital, a peri-urban health center, and a rural mission health center in Mazabuka District, and a rural health center in Monze District. All four sites provide antenatal care, well-baby care, and PMTCT services to patients free of charge. Between 740 and 1,280 pregnant women sought care at the maternal and child health departments at each study site in 2011.

### Patient Sample

At each study site, we enrolled all HIV-infected (index) mothers who: (1) made a first antenatal visit to the study site between April 1, 2011 and September 30, 2011; (2) received a positive HIV test result at the study site during the antenatal period (regardless of whether their HIV status was previously known); (3) were not already on triple-drug ART at the time of their first antenatal visit; and (4) were not known to have transferred to another site for antenatal, well-baby, or PMTCT-related care before six months after delivery. Of the 140 HIV-infected mothers screened for potential inclusion in the study, we excluded 36 mothers because they were already on triple-drug ART at the time of their first antenatal visit and five mothers because they were known to have transferred to another clinic before six months after delivery. We enrolled the remaining 99 HIV-infected mothers in the index cohort. For each index mother, we enrolled an HIV-uninfected (comparison) mother who made a first antenatal visit to the study site in the same calendar month and had a gestational age within four weeks of the index mother. All babies born to enrolled index and comparison mothers were also enrolled in the study.

### Patient Data Collection

Data on outcomes and resources utilized by each mother/baby pair for antenatal, well-baby, and PMTCT-related care, from the first antenatal visit through six months after delivery, were obtained from site registers. Mother/baby pairs were tracked longitudinally through eight standard registers and multiple improvised registers at each site using site-assigned mother and baby identification numbers. The eight standard registers tracked key components of the antenatal, well-baby, and PMTCT care continuum, including: (1) antenatal and postnatal care for all mothers; (2) HIV testing and counseling for all mothers and PMTCT-related care for HIV-infected mothers; (3) delivery information for all mothers; (4) additional delivery information for HIV-infected mothers and HIV-exposed babies; (5) well-baby care for all babies; (6) PMTCT-related care for HIV-exposed babies; (7) lay counselor interactions with HIV-infected mothers and HIV-exposed babies; and (8) receipt of HIV DNA PCR test results for HIV-exposed babies. The improvised registers, used by the sites to track information not captured in detail in the formal registers, varied by site and included PMTCT-related drug dispensing information for HIV-infected mothers and their babies, information on HIV re-testing for mothers who previously received a negative HIV test result, drug dispensing information for the treatment of syphilis, and additional tracking information for HIV DNA PCR tests and rapid HIV tests.

### Classification of Patient Outcomes

Each mother/baby pair was assigned two outcomes, one based on the baby’s status at the time of delivery and one based on the baby’s status six months after delivery. At the time of delivery, a baby was classified as *known to have died* if a death before or during delivery was indicated in the site registers. A baby was classified as *lost to follow up* if they were not known to have died before or during delivery but did not have a visit to the study site at or after delivery. Six months after delivery, a baby was classified as *known to have died* if the baby had a known live birth and a death was indicated in the site registers between the time of delivery and six months after delivery. A baby was classified as *lost to follow up* if they were not known to have died but did not have a visit to the study site during months five through seven after delivery. All babies not classified as *known to have died* or *lost to follow up* at either time point were classified as *retained in care*. Differences in patient outcomes between the index and comparison groups were calculated using Pearson’s chi-squared test. A p-value of less than 0.05 was considered statistically significant.

HIV-exposed babies retained in care six months after delivery were further categorized based on HIV status. A baby was classified as *HIV infected* if they had a dried blood spot sample collected within seven months of delivery that was HIV positive. A baby was classified as *HIV uninfected* if they had a dried blood spot sample collected during months five through seven after delivery that was HIV negative. Because dried blood spot samples are sent to a centralized laboratory in Lusaka for HIV DNA PCR testing, there is a delay between the time when a dried blood spot sample is collected and when HIV test results are reported back to the study site. Babies were categorized as *HIV infected* or *HIV uninfected* based on the date when the dried blood spot sample was collected and not the date when the test result was reported back to the study site. All HIV-exposed babies not classified as *HIV-infected* or *HIV-uninfected* were classified as *HIV status unknown*.

### Unit Costs

The costs of all fixed and variable site- and district-level resources used by the study sites to provide antenatal, well-baby, and PMTCT services to study subjects were included, regardless of funding source. Fixed resources included buildings, equipment, and support staff. Variable resources included ARV drugs used for prophylaxis, non-ARV drugs, diagnostics, vaccines, and provider time for clinic visits. Unit costs were estimated for the 2011 calendar year using financial records, interviews with site managers, national drug price lists, a schedule of government salaries and allowances, and market prices [18]. Costs reported in Zambian Kwacha (ZMK) were first adjusted to 2011 levels, if necessary, using the consumer price index and then converted to US dollars at a rate of 4,861 ZMK/$, the average exchange rate for 2011 [19], [20]. Additional details on the estimation of unit costs are provided in Appendix S1.

Unit costs for drugs, diagnostics, and vaccines were identical across sites. Per visit costs for fixed resources and provider time were site-specific but varied little across sites. In our primary analysis, we applied the per visit unit costs for fixed resources and provider time from the urban district hospital to visits at all four study sites.

We excluded costs for outpatient care above the district level (e.g., costs of oversight or training), costs for outpatient services provided to anyone other than the mother or baby (e.g., HIV test for the mother’s partner), costs for inpatient services, including delivery, and costs to the patients (e.g., clinic fees, transport). We also excluded costs for triple-drug ART services, including the cost of CD4 tests, in our primary analysis.

### Average Resource Utilization and Costs of Care

In our primary analysis, we calculated the total number of each resource utilized by each mother/baby pair. We then calculated total costs for each mother/baby pair by multiplying the unit cost for each resource by the total number of resources utilized. We calculated average resource utilization and average costs for the index and comparison cohorts by dividing total resource utilization and costs for all mother/baby pairs in each cohort by the total number of mother/baby pairs in each cohort. We repeated the same calculations for the subset of mother/baby pairs retained in care six months after delivery.

In secondary analyses, we estimated the average cost of triple-drug ART services for the mother, in addition to the costs of antenatal, well-baby, and PMTCT services, under Option A and under Option B+. To consider the additional costs of triple-drug ART services, we stratified index mother/baby pairs based on whether the mother was considered to have initiated triple-drug ART, defined as having either a CD4≤350 cells/µL or an ART referral indicated in the site registers, even if a CD4 test result was not indicated. Only mothers considered to have initiated triple-drug ART accrued costs for triple-drug ART services. Finally, to put our results into context, we estimated the total cost for hypothetical index and comparison mother/baby pairs presenting to care at a gestational age of 24 weeks, similar to patients in our sample, and receiving guideline-concordant care in Zambia through six months after delivery under both Option A and Option B+. Additional details on the estimation of triple-drug ART costs and on the quantity of each resource included in the hypothetical, guideline-concordant scenario are provided in Appendix S1.

Differences in average resource utilization and costs between mother/baby pairs in the index and comparison groups, and between mother/baby pairs within the index group who were either considered or not considered to have initiated triple-drug ART, were calculated using an independent two-sided t-test. A p-value of less than 0.05 was considered statistically significant.

## Results

### Cohort Characteristics

At their first antenatal visit, index mothers had a mean age of 27.6 years, a median gravidity of 4, and a mean gestational age of 23.6 weeks (Table 1). Compared to index mothers, comparison mothers were younger (24.2 years, p<0.001) and had a lower gravidity (3, p = 0.002) and lower gestational age (21.5 weeks, p = 0.009). Of the 99 index mothers enrolled, only 52% had a CD4 test result recorded in the site registers after HIV diagnosis; the median CD4 for those with a result recorded was 387 cells/µL. Fifty-five percent of index mothers were considered to have initiated triple-drug ART using the definition above.

**Table 1:** Baseline cohort characteristics in an evaluation of the uptake, outcomes, and costs of antenatal, well-baby, and PMTCT services under routine care conditions in Zambia.

	HIV-infected indexmothers	HIV-uninfectedcomparison mothers	p-value^a^
Sample size, n	99	99	n.a.
Mother’s age in years at first antenatal visit, mean (95% CI)	27.6 (26.5–28.8)	24.2 (23.1–25.3)	<0.001
Gravidity, median [IQR]	4 [2]–[5]	3 [1]–[4]	0.002
Gestational age in weeks at first antenatal visit, mean (95% CI)	23.6 (22.4–24.8)	21.5 (20.3–22.6)	0.009
CD4 test result recorded in site register after diagnosis, n (% of total mothers enrolled)	51 (52)	n.a.	n.a.
CD4 cell count, median cells/µL [IQR]	387 [303–519]	n.a.	n.a.
CD4≤350 cells/µL indicated in site register, n (% of total mothers enrolled)	17 (17)	n.a.	n.a.
ART referral indicated in site register, n (% of total mothers enrolled)	49 (49)	n.a.	n.a.
Mother considered to have initiated triple-drug ART due to a CD4≤350 cells/µLor an ART referral indicated in site register, n (% of total mothers enrolled)	54 (55)	n.a.	n.a.

ART: antiretroviral therapy; CI: confidence interval; IQR: interquartile range; PMTCT: prevention of mother-to-child transmission.

a Differences in means were calculated using an independent two-sided t-test. Differences in medians were calculated using the Wilcoxon rank sum test.

### Patient Outcomes

Outcomes at delivery and six months after delivery are presented in Table 2. Retention in care was significantly higher for index babies than for comparison babies at both time points. At delivery, 81% of index babies were retained in care, compared to 53% of comparison babies (p<0.001). By six months after delivery, 62% of index babies were retained in care, compared to only 30% of comparison babies (p<0.001). Of the index babies retained in care six months after delivery, 3% were known to be HIV infected, 30% were known to be HIV uninfected, and 67% had an unknown HIV status. Of the babies retained in care at delivery, 70% of index babies and 85% of comparison babies were known to have been delivered in a health facility (p = 0.056).

**Table 2:** Baby retention in care at the time of delivery and six months after delivery.

Outcome, n (%)	HIV-exposed indexbabies n = 99	HIV-unexposed comparisonbabies n = 99	p-value^a^
**At the time of delivery**			
Retained in care	80 (81)	52 (53)	<0.001
* Known to have been delivered in a health facility*	*56 (70)*	*44 (85)*	*0.056*
Known to have died^b^	7 (7)	3 (3)	0.194
Lost to follow up	12 (12)	44 (44)	<0.001
**Six months after delivery**			
Retained in care	61 (62)	30 (30)	<0.001
* HIV exposed, HIV infected*	*2 (3)*	*n.a.*	*n.a.*
* HIV exposed, HIV uninfected*	*18 (30)*	*n.a.*	*n.a.*
* HIV exposed, HIV status unknown*	*41 (67)*	*n.a.*	*n.a.*
Known to have died	8 (8)	3 (3)	0.121
Lost to follow up	30 (30)	66 (67)	<0.001

HIV: human immunodeficiency virus.

a Differences in proportions were calculated using Pearson’s chi-squared test.

b Babies known to have died at the time of delivery include babies known to have died before or during delivery.

### Resource Utilization and Unit Costs

The quantities of resources utilized for the provision of antenatal, well-baby, and PMTCT-related care and the unit costs for these resources are reported in Tables 3 and 4. During the antenatal period, comparison mothers averaged 3.0 visits to the study sites and index mothers averaged 3.2 visits (p = 0.528). Most other antenatal resource utilization was similar between the two groups, with the exception of HIV rapid tests (1.2 per comparison mother vs. 1.0 per index mother, p<0.001) and sulfadoxine/pyrimethamine tablets (5.4 vs. 3.7 tablets, p<0.001).

**Table 3:** Quantity of resources utilized and unit costs for the provision of antenatal and well-baby services from the first antenatal visit through six months after delivery.

	Resource utilization for all mother/baby pairs in sample			Resource utilization for mother/baby pairsretained in care through 6 months after delivery				Unit cost(2011 USD)
	Index mother/baby pairs	Comparison mother/baby pairs	p-value^a^	Index mother/baby pairs	Comparisonmother/baby pairs	p-value^a^		
Number of mother/baby pairs, n	99	99	–	61	30	–		–
**Antenatal care**								
Gestational age in weeks at first antenatal visit, mean	23.6	21.5	0.009	24.8	23.3	0.166		–
Months of antenatal follow up time, mean^b^	3.3	3.6	0.161	3.5	3.9	0.160		–
Outpatient clinic visits, mean^c^	3.2	3.0	0.528	3.5	3.2	0.363		$4.01/visit^g^
Hemoglobin tests, mean^c^	0.7	0.7	1.000	0.8	0.9	0.571		$1.03/test
Rapid plasma reagin tests, mean^c^	0.5	0.5	0.777	0.4	0.6	0.212		$0.20/test
Urine dipstick tests, mean^c^	0.2	0.2	0.866	0.2	0.4	0.031		$0.17/test
Ultrasounds, mean^c^	0.0	0.0	0.414	0.0	0.0	0.609		$5.45/scan
First HIV rapid tests, mean^cd^	1.0	1.2	<0.001	1.0	1.4	<0.001		$0.90/test
Confirmatory HIV rapid tests, mean^c^	1.0	0.0	<0.001	1.0	0.0	<0.001		$1.86/test
Tetanus toxoid vaccine doses, mean^c^	0.5	0.6	0.697	0.5	0.6	0.614		$0.59/dose
Ferrous sulfate 200 mg tablets, mean^cd^	63.0	70.3	0.112	71.3	81.0	0.127		<$0.01/tablet
Folic acid 5 mg tablets, mean^cd^	56.5	62.9	0.164	61.1	65.5	0.510		<$0.01/tablet
Multivitamin tablets, mean^c^	6.4	7.6	0.599	5.4	2.0	0.184		$0.24/tablet
Sulfadoxine/pyrimethamine 500 mg/25 mg tablets, mean^cd^	3.7	5.4	<0.001	3.9	6.5	<0.001		$0.02/tablet
Mebendazole 500 mg tablets, mean^c^	0.8	0.9	0.141	0.8	0.9	0.279		$0.02/tablet
Benzathine penicillin 2.4 MU doses, mean^c^	0.1	0.0	0.085	0.1	0.0	0.258		$0.37/dose
**Well-baby care**								
Months of follow up time after delivery, mean^e^	4.0	2.1	<0.001	5.7	5.6	0.546	–	
Outpatient clinic visits, mean^f^	4.9	2.5	<0.001	7.0	6.6	0.136	$4.01/visit^g^	
BCG vaccine doses, mean^f^	0.6	0.4	0.001	0.9	0.9	0.476	$0.10/dose	
DPT-HepB-Hib vaccine doses, mean^f^	1.6	1.0	0.003	2.3	2.7	0.031	$2.99/dose	
Oral polio vaccine doses, mean^f^	2.1	1.3	0.002	3.1	3.6	0.026	$0.13/dose	
Vitamin A 200,000 IU gel caps, mean^f^	0.0	0.0	0.563	0.0	0.0	0.989	$0.01/gel cap	

BCG: Bacille Calmette Guerin; DPT-HepB-Hib: diphtheria, pertussis, tetanus, hepatitis B, haemophilus influenza type b; HIV: human immunodeficiency virus; USD: United States dollar.

a Differences in means were calculated using an independent two-sided t-test.

b Mean time from the first antenatal visit to delivery for mother/baby pairs retained in care through delivery and mean time from first antenatal visit to last antenatal visit for mother/baby pairs not retained in care through delivery.

c Zambian national guidelines recommend the following for a pregnant woman presenting to care with a gestational age of 24 weeks: four outpatient clinic visits; one hemoglobin test and one urine dipstick test at the first antenatal visit; one hemoglobin test at a subsequent antenatal visit for HIV-infected women; two rapid plasma reagin tests, one at the first visit and one three months later; one rapid HIV test at the first antenatal visit with a second, confirmatory rapid HIV test if the first rapid HIV test is positive or with repeat rapid HIV tests every three months during pregnancy and while breastfeeding if the first rapid HIV test is negative; two doses of tetanus toxoid vaccine four weeks apart for pregnant women who have not been previously vaccinated and one dose of tetanus toxoid vaccine for pregnant women who have been previously vaccinated and who have received less than five previous doses in total; daily supplements (30 tablets per month) of ferrous sulfate 200 mg tablets and folic acid 5 mg tablets; three doses (nine tablets) of sulfadoxine 500 mg/pyramethamine 25 mg for HIV-uninfected pregnant women; four tablets of mebendazole 500 mg, one at each antenatal visit; and one 2.4 MU dose of benzathine penicillin for women with a positive rapid plasma reagin test [5].

d Small quantities of the following resources were utilized by mothers in our sample during the postnatal period rather than the antenatal period: first rapid HIV tests (3 tests in total), ferrous sulfate 200 mg (120 tablets), folic acid 5 mg (104 tablets), and sulfadoxine 500 mg/pyramethamine 25 mg (6 tablets). This resource utilization is included in the average resource utilization figures in Table 3.

e Mean time from delivery to the last well-baby visit within six months after delivery. Patients with no visits after delivery had zero months of postnatal follow up.

f Zambian national guidelines recommend the following care for babies during the first six months after delivery: seven outpatient clinic visits; one dose of the BCG vaccine at birth (with a repeat dose at 12 weeks of age if the infant does not have a scar); four doses of the OPV vaccine at birth, six weeks, 10 weeks, and 14 weeks; three doses of DPT-HepB-Hib at six weeks, 10 weeks, and 14 weeks; and a one-time vitamin A supplement of 100,000 IU (half of a 200,000 IU Vitamin A gel cap) at six months [5].

g The cost per outpatient clinic visit of $4.01 is for the urban district hospital and includes $2.46 per visit for provider time and $1.55 per visit for fixed resources. In the primary analysis, the unit cost for the urban district hospital was applied to outpatient visits at all four study sites. The cost per outpatient clinic visit, including the cost of fixed resources and provider time, was $2.42 at the peri-urban health center, $4.45 at the rural mission health center, and $2.56 at the rural health center.

**Table 4:** Quantity of resources utilized and unit costs for the provision of PMTCT services from the first antenatal visit through six months after delivery.

	Resource utilization for all index mother/baby pairs in sample				Resource utilization for index mother/baby pairs retained in care through 6 months after delivery				Unit cost(2011 USD)
	All mother/baby pairs	Subset with motherconsidered to haveinitiated triple-drugART^a^	Subset withmother notconsidered tohave initiatedtriple-drug ART^a^	p-value^b^	All mother/baby pairs	Subset with motherconsidered to haveinitiated triple-drugART^a^	Subset withmother notconsidered tohave initiatedtriple-drug ART^a^	p-value^b^	
Number of mother/baby pairs, n	99	54	45	–	61	33	28	–	–
**Resources utilized by mother** c									
Co-trimoxazole 400 mg/80 mg tablets, mean	50.0	32.2	71.3	0.011	57.5	35.5	84.6	0.012	$0.02/tablet
AZT 300 mg tablets, mean	80.6	69.4	94.0	0.153	90.5	75.5	108.2	0.155	$0.12/tablet
3TC 150 mg tablets, mean	4.8	4.1	5.6	0.284	6.2	5.1	7.5	0.183	$0.04/tablet
NVP 200 mg tablets, mean	0.4	0.4	0.5	0.558	0.6	0.5	0.6	0.479	$0.04/tablet
**Resources utilized by baby** d									
Co-trimoxazole 240 mg/5 ml suspension100 ml bottles, mean	2.8	2.2	3.6	0.029	4.4	3.4	5.6	0.005	$0.33/bottle
NVP 10 mg/ml suspension 25 ml bottles, mean	1.8	1.3	2.5	0.026	2.8	1.8	4.1	0.003	$2.31/bottle
HIV DNA PCR test, mean	0.6	0.6	0.8	0.096	0.9	0.8	1.1	0.022	$13.88/test

3TC: lamivudine; ARV: antiretroviral; AZT: zidovudine; DNA: deoxyribonucleic acid; HIV: human immunodeficiency virus; NVP: nevirapine; PCR: polymerase chain reaction; PMTCT: prevention of mother-to-child transmission; USD: United States dollar.

a A mother was considered to have initiated triple-drug ART if site registers indicated that she had either a CD4≤350 cells/µL or an ART referral indicated in the site registers.

b Differences in means between mothers considered to have initiated triple-drug ART and not considered to have initiated triple-drug ART were calculated using an independent two-sided t-test.

c Zambian national guidelines recommend co-trimoxazole 400 mg/80 mg tablets twice daily from 14 weeks gestation for all HIV-infected pregnant women [5]. The guidelines also recommend ARV prophylaxis for HIV-infected pregnant women not yet on triple-drug ART, including: AZT 300 mg tablets twice daily from 14 weeks gestation through one week postpartum, one NVP 200 mg tablet at delivery, and 3TC 150 mg tablets twice daily from delivery through one week postpartum [5].

d Zambian national guidelines recommend co-trimoxazole prophylaxis for HIV-exposed babies from six weeks of age until HIV infection is excluded, with a recommended dose of 2.5 ml of 240 mg/5 ml co-trimoxazole suspension per day for babies less than six months of age [5]. The guidelines also recommend daily NVP from birth through one week after the cessation of breastfeeding for infants born to mothers not yet on triple-drug ART and daily NVP from birth through six weeks of age for infants born to mothers on triple-drug ART, with a recommended dose of 1–1.5 ml of 10 mg/ml NVP suspension per day from birth to six weeks and 2 ml per day from six weeks to six months of age [5]. The guidelines also recommend that HIV-exposed infants receive a first HIV DNA PCR test at 6 weeks of age and a second HIV DNA PCR test at six months of age if the first HIV DNA PCR test was negative [5].

During the first six months after delivery, comparison mother/baby pairs averaged 2.5 visits to the study sites, significantly less than index mother/baby pairs who averaged 4.9 visits to the study sites (p<0.001). Comparison babies received significantly fewer doses of the BCG vaccine (0.4 vs. 0.6 doses, p = 0.001), the DPT-HepB-Hib vaccine (1.0 vs. 1.6 doses, p = 0.003), and the oral polio vaccine (1.3 vs. 2.1 doses, p = 0.002). These differences largely result from the difference between the two groups in the proportions retained in care.

As would be expected, index mother/baby pairs with mothers considered to have initiated triple-drug ART utilized fewer PMTCT-related resources, including zidovudine, lamivudine, and nevirapine prophylaxis for the mother and nevirapine prophylaxis for the baby, than pairs with mothers not considered to have initiated triple-drug ART.

### Average Cost Per Mother/Baby Pair Based on Actual Care Received

The average cost per index mother/baby pair for antenatal, well-baby, and PMTCT services, from the first antenatal visit through six months after delivery, was $69 (Table 5). The average cost per index mother/baby pair with a mother considered to have initiated triple-drug ART was $64. The average cost per comparison mother/baby pair, at $31, was significantly less than the average cost per index mother/baby pair (p<0.001).

**Table 5:** Average cost per mother/baby pair for actual antenatal, well-baby, and PMTCT services received and estimated triple-drug ART services received from the first antenatal visit through six months after delivery.

	Average cost for all mother/baby pairs in the sample					Average cost for mother/baby pairs retained in care through 6 months after delivery				
	Index mother/baby pairs			Comparison mother/baby pairs	p-value^b^	Index mother/baby pairs			Comparison mother/baby pairs	p-value^b^
	All mother/baby pairs	Subset with mother considered to have initiated triple-drug ART^a^	Subset with mother not considered to have initiated triple-drug ART^a^			All mother/baby pairs	Subset with mother considered to have initiated triple-drug ART^a^	Subset with mother not considered to have initiated triple-drug ART^a^		
Number of mother/baby pairs, n	99	54	45	99	–	61	33	28	30	–
Cost per mother/baby pair in 2011 USD, not including ART, mean (95% CI)	$69 ($62–$76)	$64 ($55–$73)	$74 ($63–$86)	$31 ($27–$34)	<0.001	$88 ($82–$94)	$79 ($71–$87)	$98 ($89–$107)	$52 ($50–$55)	<0.001
Cost per mother/baby pair in 2011 USD, including ART, mean (95% CI)	$148($129–$168)	$210 ($187–$234)	$74 ($63–$86)	$31 ($27–$34)	<0.001	$185($163–$208)	$260($246–$273)	$98($89–$107)	$52 ($50–$55)	<0.001
Breakdown of average cost per mother/baby pair in 2011 USD by type of care, mean										
Antenatal services (actual)^c^	$19	$19	$19	$17	0.080	$20	$20	$21	$17	0.041
Well-baby services (actual)^d^	$25	$25	$25	$13	<0.001	$35	$34	$37	$35	0.843
PMTCT services (actual)^e^	$24	$20	$30	n.a.	n.a.	$32	$25	$40	n.a.	n.a.
Triple-drug ART services (estimated)^f^	$80	$146	n.a.	n.a.	n.a.	$98	$180	n.a.	n.a.	n.a.

ART: antiretroviral therapy; CI: confidence interval; PMTCT: prevention of mother-to-child transmission; USD: United States dollar.

a A mother was considered to have initiated triple-drug ART if site registers indicated that she had either a CD4≤350 cells/µL or an ART referral indicated in the site registers.

b Differences in means between index and comparison mother-baby pairs were calculated using an independent two-sided t-test.

c Antenatal service costs include the costs of fixed resources and provider time per clinic visit, diagnostics, vaccines, and non-ARV drugs provided to both index and comparison mothers.

d Well-baby service costs include the costs of fixed resources and provider time per clinic visit, vaccines, and non-ARV drugs provided to both index and comparison babies.

e PMTCT service costs include the costs of ARV prophylaxis for index mothers not yet on triple-drug ART, ARV prophylaxis for babies, co-trimoxazole prophylaxis for index mothers and babies, and HIV DNA PCR tests for index babies.

f Triple-drug ART service costs include the costs of pre-ART and on-ART services, including costs of fixed resources, provider time for clinic visits, ARV drugs, non-ARV drugs, and diagnostics, for index mothers considered to have initiated triple-drug ART. See Appendix S1 for details on the estimate of triple-drug ART service costs.

For the subset retained in care six months after delivery, the average cost per index mother/baby pair was $88 while the average cost per index mother/baby pair with a mother considered to have initiated triple-drug ART was $79. The average cost of antenatal and well-baby services together was similar for index and comparison mother/baby pairs retained in care ($56 vs. $52), with PMTCT services costing an additional $32 for index mother/baby pairs.

Including an estimated cost for triple-drug ART services increased the average cost for the subset of mother/baby pairs with a mother considered to have initiated triple-drug ART from $64 to $210, resulting in an increase in the average cost for all index mother/baby pairs from $69 to $148. For index mother/baby pairs retained in care six months after delivery, the average cost for the subset with a mother considered to have initiated triple-drug ART increased from $79 to $260, resulting in an increase in the average cost for all index mother/baby pairs from $88 to $185. The additional cost of triple-drug ART services for mothers considered to have initiated triple-drug ART who were retained in care six months after delivery was $180, more than twice the cost of antenatal, well-baby, and PMTCT services combined.

### Cost of Guideline-concordant Care

The results reported above and in Table 5 are based on the actual quantities of resources received by index and comparison mother/baby pairs through six months after delivery. Using the same unit costs as in that analysis, we also estimated the costs of service delivery for hypothetical index and comparison mother/baby pairs presenting to care at a gestational age of 24 weeks, remaining in care through six months after delivery, and receiving the quantity of resources recommended in Zambian national guidelines [5].

The estimated cost for a hypothetical comparison mother/baby pair receiving guideline-concordant care was $60, 15% higher than the average cost of $52 for comparison mother/baby pairs retained in care six months after delivery in our cohort (Table 6). The estimated cost for a hypothetical index mother/baby pair receiving guideline-concordant care was $303 if the mother was eligible to initiate triple-drug ART and $152 if the mother was not yet eligible to initiate ART. If roughly 55% of index mother/baby pairs were eligible to initiate triple-drug ART under Option A, as estimated in our sample, the projected cost for a hypothetical index mother/baby pair under Option A was $235, 27% higher than the average cost of $185 for index mother/baby pairs in our cohort.

**Table 6:** Estimated cost for hypothetical mother/baby pairs presenting to care at a gestational age of 24 weeks and receiving guideline-concordant care from the first antenatal visit through six months after delivery^a^.

	Index mother/baby pair		Comparison mother/baby pair
	Mother eligible toinitiate triple-drugART	Mother not yet eligibleto initiate triple-drugART	
Projected cost for hypothetical mother/baby pair, 2011 USD	$303	$152	$60
Breakdown of projected cost for hypothetical mother/babypair by type of care, 2011 USD			
Antenatal services	$22	$22	$23
Well-baby services	$38	$38	$38
PMTCT services	$40	$92	n.a.
Triple-drug ART services	$203	n.a.	n.a.

ART: antiretroviral therapy; PMTCT: prevention of mother-to-child transmission; USD: United States dollar.

a See Appendix S1 for details on the calculation of the projected cost for mother/baby pairs receiving guideline-concordant care.

### Anticipated Costs under Option B+

Under Option B+, all HIV-infected mothers are eligible for lifelong ART. If 100% of index mothers enrolled in our study had been ART eligible, we would expect an average actual cost per index mother/baby pair retained in care six months after delivery of $260 instead of $185. The projected cost for a hypothetical index mother/baby pair receiving guideline-concordant care under Option B+ would be $303, 64% higher than the estimated average cost of $185 under Option A.

## Discussion

In this study, we evaluated the delivery of antenatal, well-baby, and PMTCT services under routine care conditions after the adoption of Option A in Zambia. Better information about the uptake, outcomes, and costs of service delivery will contribute both to understanding the strengths and weaknesses of Option A and to planning for the shift to Option B+ that is now underway [6].

We found that twice as many index as comparison mother/baby pairs remained in care six months after delivery. Likely as a result, HIV-exposed babies were significantly more likely to receive early infant vaccinations than were the babies of HIV-uninfected mothers. Both comparison and index mother/baby pairs utilized fewer resources than would have been utilized if they were receiving fully guideline-concordant care. The average cost per comparison mother/baby pair retained in care was $52, compared to an expected cost of $60 for guideline-concordant antenatal and well-baby care. The average cost per index mother/baby pair retained in care was $88, including $56 for antenatal and well-baby care and $32 for PMTCT-related care. Adding an estimated cost for triple-drug ART services increased the average cost per index mother/baby pair retained in care to $185, compared to an expected cost of $235 for guideline-concordant care under Option A. If 100% of index mothers were eligible to initiate ART, as will be the case under Option B+, the average actual cost per mother/baby pair would be $260, compared to an expected cost of $303 for guideline-concordant care.

Guideline-concordant uptake of antenatal, well-baby, and PMTCT services requires that women present to antenatal care early in their pregnancy, that they remain in care through the cessation of breastfeeding, and that they receive regular services throughout their time in care. Our findings suggest several areas where guideline concordance, and consequently the quality of care, for mother/baby pairs could be improved. First, index mothers are presenting to antenatal care late in their pregnancy. This leaves less time for them to receive critical laboratory tests, drugs, and vaccines for their health and the health of their baby prior to delivery. Observational data suggest that earlier presentation to care in pregnancy, if it results in earlier initiation of ARV prophylaxis and ART, could reduce the risk of vertical transmission [21].

Second, mother/baby pairs are falling out of care rapidly. Only 81% of index mother/baby pairs and 53% of comparison mother/baby pairs were retained in care at the time of delivery, and only 62% of index mother/baby pairs and 30% of comparison mother/baby pairs were retained in care six months after delivery.

Third, ascertainment of HIV status for HIV-exposed babies is poor. Of the index babies retained in care, the majority (67%) had an unknown HIV status six months after delivery. Adding on to this 67% the number of babies whose HIV status remained unknown because they were not retained in care, these data suggest that the HIV status for the vast majority of HIV-exposed infants is unknown at six months after delivery. Timely and comprehensive ascertainment of HIV status for HIV-exposed babies is critical for being able to assess the performance of any PMTCT program. It is also critical for ensuring early identification and effective linkage to triple-drug ART services for HIV-infected infants, which can increase their chances of survival [22].

Fourth, recordkeeping for mothers and babies, based on a system of multiple registers, is disjointed, ambiguous, and incomplete, making it difficult to determine whether HIV-infected mothers and their HIV-exposed babies are receiving the care that they should be receiving for PMTCT. Eligibility for triple-drug ART can be ascertained from two fields in the site registers. The first field indicates the date when a patient was assessed for triple-drug ART eligibility and the result of the assessment in terms of CD4 or WHO stage. Only 52% of index mothers had a CD4 test result recorded in the site registers after diagnosis of HIV; no index mothers had a WHO stage recorded. The second field, which indicates whether and when a mother was referred for triple-drug ART, is completed inconsistently across sites. No fields in the site registers capture a confirmation of triple-drug ART initiation. While 55% of index mothers in our sample appear to have been eligible for triple-drug ART after their first antenatal visit based on CD4 test results or ART referral, there is no way to know whether they initiated triple-drug ART and when based on the site registers. Information on ARV prophylaxis dispensed, if recorded at all, was recorded in improvised registers at each site, resulting in inconsistent records that are likely incomplete.

Shortcomings in recordkeeping aside, our data suggest that mother/baby pairs are not receiving the complete recommended package of services for PMTCT or for antenatal and well-baby care. Improving retention in care, increasing guideline concordance, and moving from Option A to Option B+ in Zambia will all increase costs in the short term, though over time could diminish the need for pediatric triple-drug ART and help improve the health of Zambian mothers and babies overall, thereby ultimately saving resources for the Zambian healthcare system.

The cost for guideline-concordant antenatal and PMTCT service delivery under Option A for a hypothetical index mother/baby pair not yet eligible to initiate triple-drug ART in the study sites is $114. This figure is consistent with the $108–$120 per patient (in 2008 US dollars) for guideline-concordant care reported in the only other published study estimating costs of antenatal and PMTCT service delivery in Zambia, which was based on guidelines prior to the adoption of Option A [10]. Because the majority of mother/baby pairs in our sample did not receive all of the care recommended by guidelines, the actual cost of antenatal and PMTCT service provision was substantially less than $114.

Our findings on uptake of antenatal and PMTCT services are consistent with previously published figures from Zambia [8], [13]. In a review of routine medical records for 115,552 pregnant women receiving antenatal care between 2007 and 2010, the median gestational age at first antenatal visit was 23 weeks [13]. Approximately 80% of women diagnosed with HIV during pregnancy received a CD4 test, of which half had a CD4≤350 cells/µL. In a review of site-level service data for 14,815 HIV-infected pregnant women receiving antenatal care in 2007 and 2008, 11% of women had a CD4 test result available with a median CD4 of 366 cells/µL; 47% of women had a CD4≤350 cells/µL [8].

Our study has several limitations. First, results are for a small number of patients at only four purposively selected sites. Because sites were not selected randomly, our findings are not representative of antenatal, well-baby, and PMTCT service delivery in Zambia as a whole. Further, because sites were selected only after confirming that patients could be traced longitudinally through the site registers, sites in our sample may have better recordkeeping than the average site. To the extent that better recordkeeping is associated with better quality care, the sites in our study may provide a more optimistic picture of antenatal, well-baby, and PMTCT service delivery than the average site in Zambia.

Second, we excluded mothers already on triple-drug ART at the time of their first antenatal visit. Because the primary objective of this study was to evaluate service delivery and costs for pregnant women newly identified with HIV infection, these women were logically excluded from the analysis. We also excluded the few mothers known to have transferred to another site during the study follow up period and excluded, by default, any mothers who did not seek antenatal care at all. These patients are likely to differ systematically from the study population.

Third, patient outcome and resource utilization results in this analysis are limited to what could be ascertained from a retrospective review of site registers. Visits to the site or resources utilized that were not recorded in the site registers have not been captured. For mother/baby pairs no longer attending the study clinic, we could not distinguish between those who had an unrecorded transfer to another clinic or an unrecorded death and those who were lost to follow up.

Finally, we excluded from our analysis the costs of the following resources: inpatient care, including delivery at a facility; outpatient services provided outside of the maternal and child health department at each site, with the exception of triple-drug ART services; outpatient services provided to the mother’s partner such as HIV testing; program management above the district level; and resources procured by individual patients, such as transport to the clinic. Average costs reported here are therefore an underestimate of the total per patient cost to Zambia of providing antenatal, well-baby, and PMTCT services.

Despite these limitations, the study’s core findings about the uptake, outcomes, and costs of antenatal, well-baby, and PMTCT services under routine care conditions provide useful information for guiding the development of maternal and child health care in Zambia and other countries weighing the benefits and costs of different strategies for improving public health. In addition to providing accurate and up to date cost estimates that can be used for budgeting and planning, the study findings illustrated a potentially important paradox of HIV care in low-income countries. In our sample, babies of HIV-infected mothers were far more likely to remain in care and receive appropriate non-HIV, well-baby care in the six months after delivery than were babies of HIV-uninfected mothers. Though the study could not describe the specific reasons for this, it touches upon one of the important debates about healthcare and HIV service delivery in resource-constrained settings, that of whether the influx of resources for HIV care strengthens or weakens other primary healthcare service delivery. In this study, there is little doubt that enrollment in a PMTCT program was associated with better well-baby care for HIV-exposed babies.

## Supporting Information

Appendix S1 **The Supplementary Appendix provides additional detail on the methods described in the main manuscript.**
(DOCX)
