# Supplementary material for: Uptake, Outcomes, and Costs of Antenatal, Well-Baby, and Prevention of Mother-to-Child Transmission of HIV Services under Routine Care Conditions in Zambia
Source: PLoS One. 2013 Aug 28;8(8):e72444. doi: 10.1371/journal.pone.0072444 (PMC3756060; doi:10.1371/journal.pone.0072444)
Supplement: Appendix S1 — The Supplementary Appendix provides additional detail on the methods described in the main manuscript. (DOCX) [file pone.0072444.s001.docx]

**Uptake, outcomes, and costs of antenatal, well-baby, and prevention of mother-to-child transmission of HIV services under routine care conditions in Zambia**

**Supplementary Appendix**

**METHODS**

**Unit cost estimates**

Fixed costs were defined as costs for resources used at study sites that could not be attributed directly to an individual subject’s care, such as buildings, equipment, and support staff. For equipment and buildings, upfront investment costs were estimated using a replacement cost approach. These costs were annualized using a 3% discount rate and an estimated working life (50 years for buildings, 5 years for equipment) [[1](#_ENREF_1)]. The annual cost of support staff was based on 2011 salaries and allowances. A fixed cost per visit was estimated by dividing the total annual fixed cost for the maternal and child health department at each site by the total number of patient consultations provided during the 2011 calendar year.

Variable costs were defined as costs for resources used to treat study subjects that could be attributed directly to an individual subject or visit, such as antiretroviral (ARV) drugs, non-ARV drugs, diagnostics, vaccines, and provider time for clinic visits. ARV and non-ARV drug costs and vaccine costs were based on standard Zambian Ministry of Health unit costs [[2](#_ENREF_2)]. Laboratory test costs for point-of-care tests (hemoglobin, rapid plasma reagin, urine dipstick, rapid HIV test), estimated as the sum of unit costs for test kits, reagents, and consumables, were also based on standard Zambian Ministry of Health unit costs [[2](#_ENREF_2)]. The cost of HIV DNA PCR tests for early infant diagnosis of HIV, the only non-point-of-care test utilized by our sample, was estimated as the sum of unit costs for test kits, reagents, consumables, equipment, labor, and space. Annualized laboratory equipment costs were divided by the total number of laboratory tests performed per year on each piece of equipment to estimate a per test cost. The cost of provider time per clinic visit was estimated by dividing the total cost of staff time for all providers conducting patient consultations, valued at 2011 salaries and allowances, by the total number of patient consultations provided during the 2011 calendar year.

**Average cost estimates for secondary analyses**

We estimated the average cost per index mother/baby pair in our sample if the cost of adult triple-drug antiretroviral therapy (ART) services, in addition to the costs of antenatal, well-baby, and prevention of mother-to-child transmission of HIV (PMTCT) services, was included. We used an estimated cost of $20.54 per patient-month in care for on-ART services and a cost of $7.52 per patient-month in care for pre-ART services, based on the cost per patient-month in care for on-ART services less the cost of antiretroviral drugs. The pre-ART cost per patient-month in care was applied to the first half month in care for index mothers considered to have initiated triple-drug ART; the on-ART cost per patient-month in care was applied from the second half month in care until the last visit to the study site or until six months after delivery, whichever came first. The estimated pre-ART and on-ART costs per patient-month in care are adapted from a previous study on the costs and outcomes of adult ART in Zambia [[3](#_ENREF_3)]. Costs in the previous study were estimated using methods similar to those in the current study and are in 2011 USD. The cost of pre-ART services includes the cost of non-ARV drugs, diagnostics, including CD4 tests, provider time for outpatient visits, and fixed resources. The cost of on-ART services also includes the cost of ARV drugs.

We also estimated costs for hypothetical index and a hypothetical comparison mother/baby pair who reported for their first antenatal visit with a gestational age of 24 weeks, remained in care until six months after delivery, and received guideline-concordant care under Option A. In this scenario, we included the same pre-ART and on-ART costs per patient-month in care as in the first scenario and assumed that each mother/baby pair received all resources recommended by the 2010 Zambian PMTCT guidelines [[4](#_ENREF_4)].

For antenatal care for the mother, resources included four outpatient clinic visits, one hemoglobin test for HIV-uninfected women and two hemoglobin tests for HIV-infected women, two rapid plasma reagin tests, one urine dipstick test, two rapid HIV tests (a first test and a confirmatory test) for index mothers and four rapid HIV tests (one every three months from the first antenatal visit through six months after delivery) for comparison mothers, one dose of the tetanus toxoid vaccine, daily ferrous sulfate and folic acid supplements, four mebendazole tablets for deworming, and three doses (nine tablets) of sulfadoxine/pyramethamine for malaria prophylaxis for comparison mothers. For well-baby care, resources included seven outpatient clinic visits, one dose of the BCG vaccine, three doses of the DPT-HepB-Hib vaccine, four doses of the oral polio vaccine, and a single supplement of vitamin A.

PMTCT resources utilized depended on the mother’s eligibility for triple-drug ART. For a hypothetical, index mother not yet eligible to initiate triple-drug ART, resources included twice daily co-trimoxazole from the first antenatal visit through six months after delivery, twice daily zidovudine from the first antenatal visit through one week postpartum, twice daily lamivudine from delivery through one week postpartum, and a single dose of nevirapine to be taken at delivery. For a hypothetical HIV-exposed baby born to a mother not yet eligible to initiate triple-drug ART, resources included two DNA PCR tests (one at six weeks and one at six months after delivery), daily co-trimoxazole from six weeks of age through six months after delivery, and daily nevirapine from birth through six months after delivery. For a hypothetical, index mother eligible to initiate triple-drug ART, resources included two weeks of twice daily co-trimoxazole and twice daily zidovudine and a single dose of nevirapine, all dispensed at the first antenatal visit prior to determination of eligibility for triple-drug ART, two weeks of pre-ART care, and 38 weeks of on-ART care (from two weeks after the first antenatal visit through six months after delivery). For a hypothetical HIV-exposed baby born to a mother on triple-drug ART, resources included two DNA PCR tests, daily co-trimoxazole from six weeks of age through six months after delivery, and daily nevirapine from birth through six weeks of age.

To estimate the cost of guideline-concordant care under Option A, we assumed 55% of women were eligible to initiate triple-drug ART and 45% were not yet eligible based on the women in our sample. To estimate the cost of guideline-concordant care under Option B+, we assumed 100% of women were eligible to initiate triple-drug ART.

**REFERENCES**

1. Weinstein MC, Siegel JE, Gold MR, Kamlet MS, Russell LB (1996) Recommendations of the Panel on Cost-effectiveness in Health and Medicine. JAMA 276: 1253-1258.

2. Limited MS (2010) Medical Stores Limited: 2010 catalogue. Lusaka, Zambia: Government of the Republic of Zambia, Ministry of Health.

3. Rosen S, McCoy K, Mazimba A, Hamazakaza P, Long L, et al. Outcomes and outpatient costs of differenct models of AIDS treatment delivery in Zambia [abstract MOPED014]; 5th IAS Conference on HIV Pathogenesis, Treatment and Prevention, 19-22 July 2009; Cape Town, South Africa.

4. Government of the Republic of Zambia, Ministry of Health (2010) 2010 National protocol guidelines: Integrated prevention of mother-to-child transmission of HIV. Available at: <http://www.k4health.org/sites/default/files/National%20PMTCT%20Protocol%20Guidelines.pdf>. Accessed: 13 May 2013.
